# FXR acts as a therapeutic target for ulcerative colitis via suppressing ferroptosis

**DOI:** 10.1186/s10020-025-01305-3

**Published:** 2025-07-18

**Authors:** Chenye Zhao, Xiaopeng Li, Mingchao Mu, Liyong Quan, Hang Yuan, Jianbao Zheng, Wei Zhao, Xuejun Sun, Junhui Yu

**Affiliations:** 1 Department of General Surgery, The First Affiliated Hospital of Xi’an Jiaotong University, Xi’an, Shaanxi Province 710061 China; 2 Department of Orthopedic, Xi’an Chest Hospital, Xi’an, Shaanxi Province 710100 China

**Keywords:** FXR, Colitis, Ferroptosis, SLC7A11, GPX4, OTUB1

## Abstract

**Background:**

The involvement of ferroptosis in ulcerative colitis (UC) is increasingly acknowledged. Several investigations have revealed the various mechanisms by which the farnesoid X receptor (FXR) inhibits ferroptosis in certain diseases; however, its potential modulation of ferroptosis in UC remains unexplored.

**Methods:**

The characteristics of FXR expression in colitis were evaluated in the GEO database, patient specimens, and mice with DSS-induced colitis. The role of FXR in ferroptosis was investigated by treating colitis mice with the intestine-restricted FXR agonist fexaramine (Fex) intragastrically. In vitro, Caco-2 cells challenged with RSL3 were used to study the role of FXR in modulating ferroptosis in intestinal epithelial cells (IECs).

**Results:**

Fex significantly alleviated symptoms and impeded ferroptosis in mice with DSS-induced colitis. In vitro, Fex rescued Caco-2 cells from RSL3-induced ferroptosis. Mechanistically, FXR was capable of binding to the promoter region of SLC7A11 and upregulated the transcription of SLC7A11, which is beneficial for the synthesis of GSH. Knockdown of SLC7A11 partially abrogated the therapeutic effects of Fex, albeit incompletely. Further investigations revealed that FXR can also increase the protein stability of GPX4 by upregulating the deubiquitinase OTUB1.

**Conclusion:**

This study highlights that FXR exerts therapeutic effects against colitis by antagonizing ferroptosis via transactivation of SLC7A11 and increasing GPX4 stability. These results suggest that FXR may be a therapeutic target for treating colitis by antagonizing ferroptosis.

**Graphical Abstract:**

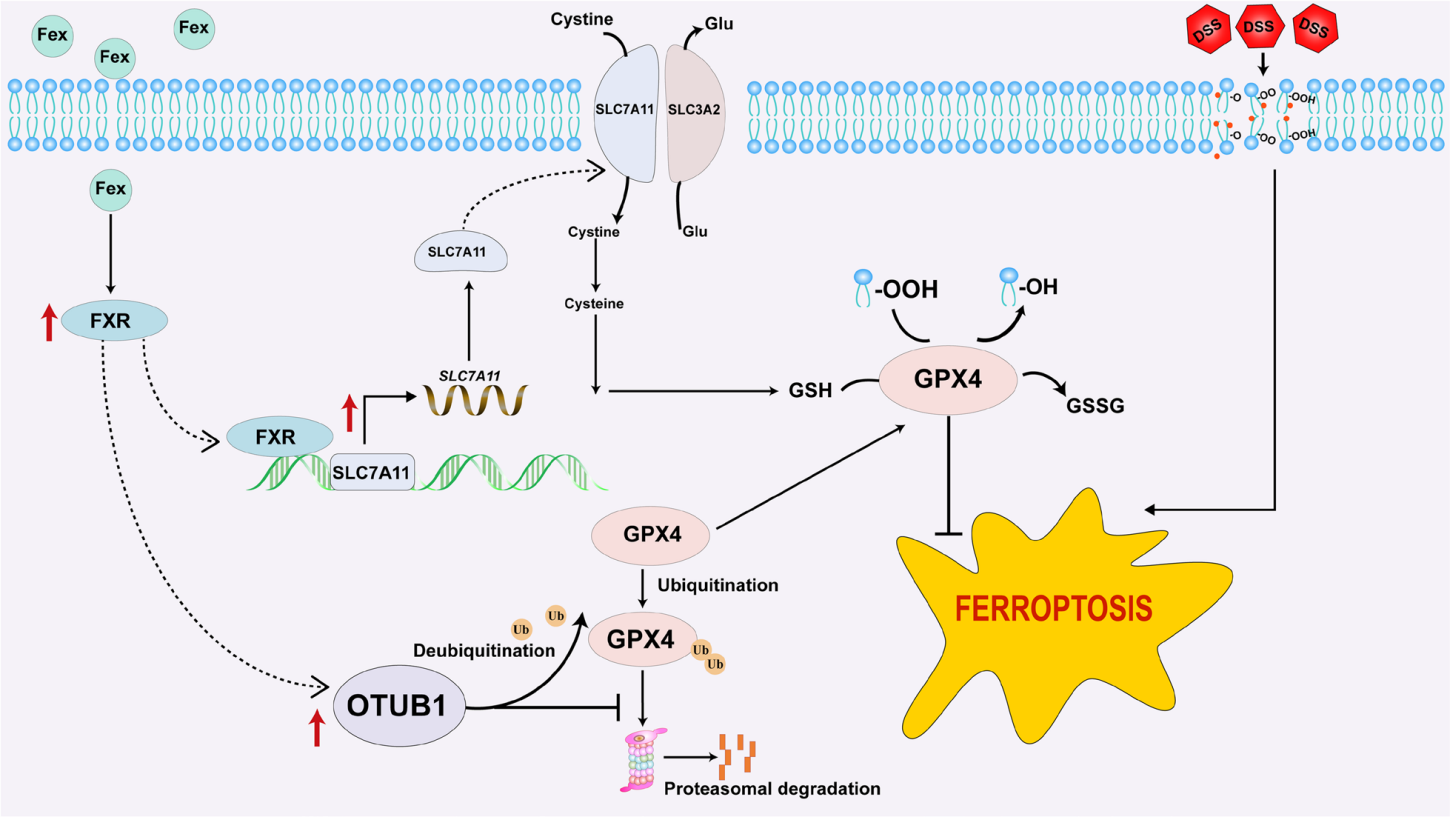

**Supplementary Information:**

The online version contains supplementary material available at 10.1186/s10020-025-01305-3.

## Introduction

Inflammatory bowel disease (IBD), which primarily comprises ulcerative colitis (UC) and Crohn’s disease (CD), is a chronic, relapsing, and challenging gastrointestinal disorder. In addition to a wide spectrum of recurrent digestive symptoms, chronic prolonged intestinal inflammation caused by colitis increases the risk of malignant colorectal tumors. Elucidating the mechanisms of IBD and developing novel therapeutic strategies are highly important. Programmed cell death (PCD) processes, such as apoptosis (Kuo et al. 2019) and pyroptosis (Yuan et al. 2018), are associated with colitis. Recent studies have indicated that ferroptosis is also involved in colitis and that the inhibition of ferroptosis may mitigate the severity of colitis (Xu et al. 2020). Nevertheless, the mechanism underlying ferroptosis regulation in colitis has not been elucidated.

Ferroptosis is a new type of iron-dependent cell death identified by Dixon et al. (2012). Multiple diseases, such as cancer, neurotoxicity, acute kidney failure, liver injury, and heart injury, are associated with ferroptosis (Jiang et al. 2021). The morphological features of ferroptosis are characterized by abnormal changes in mitochondrial cristae and membrane density. Systems Xc-GSH-GPX4, GCH1-BH_4_, and AIFM2-CoQ10 are the main antioxidant systems that resist ferroptosis by restricting lipid peroxidation (Tang and Kroemer 2020). SLC7A11 is a subunit of system Xc^-^, which is responsible for importing cystine and exporting glutamate. The intracellular reduction of cystine to cysteine is the limiting precursor for GSH synthesis. GPX4 utilizes GSH to reduce toxic phospholipid hydroperoxides (PLOOH) to nontoxic phospholipid alcohols (PLOH), thereby exerting its role as a central regulator of ferroptosis restraint. Studies have revealed that the inhibition of SLC7A11 results in ferroptosis of tumor cells and may inhibit tumor progression (Han et al. 2022), indicating that SLC7A11 is a potential therapeutic target in ferroptosis-related diseases. Physiologically, SLC7A11 is strictly regulated by transcriptional (Ye et al. 2014), epigenetic (Wang et al. 2019), and posttranslational (Martin and Gardner 2015) mechanisms. However, the profile and precise regulatory mechanism of SLC7A11 in colitis are still obscure.

Farnesoid X receptor (FXR, encoded by NR1H4 (nuclear receptor subfamily 1, group H, member 4)), is a type of bile acid nuclear receptor that is expressed primarily in the liver, kidney, and intestine. FXR is crucial in the metabolic homeostasis of bile acids (BAs) and the regulation of lipid and glucose metabolism under physiological conditions. In addition, studies have revealed that FXR is downregulated in colorectal cancer (CRC) and that increasing the expression of FXR could restrain CRC progression, indicating that FXR may act as a therapeutic target for CRC. Emerging studies have shown that FXR is abnormal in patients with colitis as well. In addition, recent studies have indicated that FXR is a pivotal modulator of lipid metabolism (CLifford et al. 2021) and oxidative stress (Wang et al. 2018), which are associated with ferroptosis. Nevertheless, the detailed role of FXR in colitis and ferroptosis remains unknown.

In this study, we aimed to explore the regulatory role of FXR in ferroptosis during colitis. Our findings revealed that FXR activation markedly alleviated colitis symptoms and reduced ferroptosis levels in mice. Mechanistically, we demonstrated that SLC7A11, a critical component of the ferroptosis defense pathway, is transcriptionally activated by FXR, whereas GPX4, another key regulator, is indirectly modulated by FXR. These results provide new insights into the role of FXR in mitigating colitis through the inhibition of ferroptosis and highlight its potential as a therapeutic target for treating colitis.

## Materials and methods

### Microarray data analysis

The free public database Gene Expression Omnibus (GEO) was used to explore the expression characteristics of the FXR gene. GSE75214 and GSE179285 were analyzed with the online tool GEO2R (a free tool from NCBI).

### Clinical samples

Patient tissues were obtained from the First Affiliated Hospital of Xi’an Jiaotong University from January 2019 to December 2022. Normal colonic tissues (non-IBD controls, abbreviated as “Control”) were acquired from non-IBD patients, whereas UC tissues were collected from patients diagnosed with UC. The term ‘non-IBD patients’ refers to individuals with non-inflammatory bowel diseases, including those who underwent surgical treatment for early-stage colon cancer or benign tumors/polyps in the colon (with no family history of cancer or IBD). This choice was made because it is extremely difficult to obtain colon tissues from truly healthy individuals (Zhao et al. 2023). The non-IBD control tissue samples were taken from normal tissue located more than 10 cm away from the lesion. The diagnosis of UC was made by experienced gastroenterologists based on the integration of the patient’s clinical presentations, laboratory tests, endoscopic findings, and histopathological results while ensuring the exclusion of other forms of colitis. All patients signed informed consent forms. The present study was approved by the Ethics Committee of the First Affiliated Hospital of Xi’an Jiao Tong University. The ethics approval number was LLSBPJ-2023-340.

### Reagents

Dextran sulfate sodium salt (DSS) with a molecular weight of 36,000 to 50,000 Da was purchased from MP Biomedical Company (Santa Ana, CA, USA). Fexaramine (Fex) (S6413) and RSL3 (S8155) were purchased from Selleck Chemical LLC (Houston, TX, USA). MG132 was purchased from Sigma‒Aldrich (St. Louis, MO, USA). Cycloheximide (CHX) (S7418) was procured from Selleck Chemical LLC (Houston, TX, USA).

### Mice

Six- to eight-week-old C57BL/6 female mice were purchased from the Laboratory Animal Centre of Xi’an Jiaotong University and maintained in a specific pathogen-free (SPF) facility. Other conditions included a 12-hour light/dark cycle, a temperature of 22–25 °C, and a humidity of 50–60%. All experimental procedures were approved by the Animal Care and Use Committee of the Medical School of Xi’an Jiaotong University (Ethics Approval No. XJTUAE2022-9523). FXR^−/−^ mice (C57BL/6) were generated by Cyagen Bioscience, Inc. (Guangzhou, China).

### Animal model

The chronic DSS-induced colitis model more closely reflects the relapsing-remitting course of human IBD. Therefore, to better simulate the clinical characteristics of ulcerative colitis, we utilized the chronic model in this study. A schematic overview of the experimental design is shown in Fig. 2A. Firstly, mice were raised for one week with free access to food and water to adapt to the new environment. Fifteen mice were randomly distributed into different groups for the following experiments (*n* = 5 in each group). The mice in the normal group (Control group) received normal water for 84 days throughout the entire experimental period. The other mice were administered 2.5% DSS (wt/vol) in drinking bottles for 7 days followed by 14 days of normal water as a cycle. When three cycles were performed in total, the chronic colitis model was constructed (the chronic colitis group was shortened to the “chronic UC” group). On the 64th day, the mice in the chronic UC group were randomly reallocated into two new groups: the DSS group (DSS) and the Fex group (shortened to “DSS + Fex”). The mice in the two new groups received another cycle of 2.5% DSS for 7 days and normal water for 14 days. The only difference was that mice in the DSS + Fex group received Fex (100 mg/kg BW, dissolved in 0.2% DMSO in PBS), while those in the DSS group were administered the vehicle (0.2% DMSO in PBS) daily via intragastric gavage.

To ensure the valid application of 2.5% DSS in the drinking water, the bottles were emptied, and fresh DSS solutions were supplied every two days. Body weight, stool consistency, and stool bleeding were evaluated daily according to the DAI scoring system (Wirtz et al. 2017). On the 85th day, all the mice were sacrificed following narcosis, and specific tissues were removed for the next study.

### ELISA

The levels of TNF-α, IL-1β, and IL-6 in mouse serum were determined using a TNF-α ELISA Kit (ab208348), an IL-1β ELISA Kit (ab197742), and an IL-6 (ab222503) ELISA Kit procured from Abcam. The measurements were performed following the instructions of the test kits.

### Cell culture

The human colonic adenocarcinoma cell lines Caco-2 and HEK293T used in the present study were purchased from the Shanghai Institute of Cell Biology, Chinese Academy of Sciences. Mycoplasma contamination was excluded before all the experiments. The cells were cultured in DMEM supplemented with 10% fetal bovine serum (FBS) (Gibco, Grand Island, NY), 100 units/ml penicillin, and 100 mg/ml streptomycin. The cells were maintained in a humidified incubator set with 5% CO_2_ and a temperature of 37 °C. When the confluence reached 70%, the cells were passaged for amplification. The cells were maintained at full confluence for differentiation and subjected to subsequent treatment. Caco-2 cells challenged with 15 µM RSL3 were used to investigate ferroptosis in IECs in vitro. To evaluate the role of FXR activation in vitro, cells were treated with Fex at a final concentration of 10 µM.

### Western blotting analysis

Western blotting analysis was performed according to standard procedures (Yu et al. 2019). Briefly, total protein from cell lines or tissues was collected. The concentration of total protein was detected with a BCA protein assay kit (CWBIO Biosciences, China). Equivalent amounts of total protein were separated via SDS–PAGE and transferred to PVDF membranes (Millipore, Billerica, MA, USA). ECL detection reagent (Mishu, Xi’an, China) was used to detect the proteins in the blots. Anti-occludin (ab216327), anti-claudin 1 (ab307692), anti-PTGS2 (ab179800), anti-GPX4 (ab125066), anti-xCT (ab175186), anti-HA tag (ab1424), anti-OTUB1 (ab270959) and anti-beta actin (ab8226) primary antibodies were procured from Abcam (Cambridge, UK). 4-Hydroxynonenal (4-HNE) antibody was purchased from R&D Systems (Minnesota, USA). NR1H4 (FXR) polyclonal antibody (25055-1-AP), Myc tag antibody (16286-1-AP), ubiquitin (Ub) antibody (10201-2-AP), Flag tag antibody (20543-1-AP), goat anti-rabbit IgG-HRP (SA00001-2), and goat anti-mouse IgG-HRP (SA00001-1) were purchased from Proteintech Group, Inc. (Wuhan, China).

### RNA Preparation and real-time PCR

Total RNA from cell lines or frozen tissues was isolated with TRIzol reagent (Invitrogen, Carlsbad, CA, USA). cDNA was synthesized using a PrimeScript RT Reagent Kit (TaKaRa, Osaka, Japan). Real-time PCR was performed with TB Green^®^ Premix Ex Taq™ II (TaKaRa, Osaka, Japan). The primer sequence information is listed in Supplementary Table 1. The 2^−∆∆CT^ method was used to compare the mRNA levels.

### Histology analysis and immunohistochemistry (IHC)

Colonic tissues were washed three times with ice-cold PBS following isolation from the mice. The tissues were then fixed in 4% paraformaldehyde for 48 h and embedded in paraffin. The tissue slides were stained with hematoxylin and eosin (H&E), periodic acid-Schiff (PAS), or alcian blue/periodic acid-Schiff (AB-PAS) to assess histological changes. IHC was performed on paraffin sections with the corresponding antibodies to evaluate the expression of the target proteins. Anti-xCT (DF12509) was purchased from Affinity Biosciences. The other antibodies used were the same as those employed wfor western blot analysis. All relevant processes were performed according to the manufacturer’s instructions. The histological score was calculated based on the degree of inflammation, lesion depth, lesion range, and degree of crypt damage (Li et al. 2022). The immunoreactivity score (IRS) was determined based on the extent and intensity of the staining, as described previously (Yu et al. 2019).

### Intracellular measurements of the contents of GSH, MDA, and Fe

Fex dissolved in DMSO was added to the culture medium at a final concentration of 10 µM, and the control group was treated with the same volume of DMSO (vehicle). After 24 h, the cells were harvested to measure the levels of GSH, Fe, and MDA and the total protein concentration. A GSH Assay Kit (A-006-2−1) and an Iron Assay Kit (A039-2-1) obtained from Nanjing Jiancheng Bioengineering Institute (Nanjing, China) were used to measure the levels of GSH and total Fe. An MDA Assay Kit(S0131S) was procured from Beyotime Biotechnology (Shanghai, China). All the experimental steps were performed following the instructions. The concentration of total protein was quantified via the BCA method as described above. The measurement results were normalized to the total protein concentration. The contents of GSH, MDA and Fe in the colonic tissues were measured as instructed in the manual.

### Cell viability

The cells were cultured with DMSO, RSL3, or RSL3 + Fex for 24 h. A Cell Counting Kit-8 (CCK8, CAT#GK10001, GLPBIO Company) was subsequently used to assay cell viability following the manufacturer’s instructions.

### ROS and lipid peroxidation measurements

To detect the effect of Fex on intracellular cellular ROS levels, a Reactive Oxygen Species Assay Kit (S0033S) was purchased from Beyotime Biotechnology. The entire process was performed according to the supplier’s instructions. Briefly, the cells were subjected to specific treatments, and then, serum-free medium supplemented with DCFH-DA (a crucial ingredient of the kit that reacts with ROS) was added to the cells, which were incubated for twenty minutes at 37 °C in the dark. The cells were washed with serum-free cell culture medium 3 times and trypsinized into single cells for the detection of ROS via flow cytometry (FCM) with 488 nm excitation and 525 nm emission. Lipid peroxidation was evaluated using C11-BODIPY581/591. Upon disposal, the cells were incubated with 2.5 µM C11-BODIPY 581/591 (GLPBIO Technology LLC) for 30 min, and the nuclei were labeled with Hoechst 33258 (final concentration of 10 µg/mL; Beijing Solarbio Science & Technology Co., Ltd.) for 20 min. After being washed with Hank’s balanced salt solution (HBSS) 3 times, the cells in HBSS were subjected to fluorescence microscopy for lipid peroxidation evaluation. Fluorescence signals from Hoechst and oxidized and nonoxidized C11 BODIPY 581/591 were acquired in the 460 nm (blue), 510–550 nm (green), and 585–591 nm (red) channels, respectively.

### Transmission electron microscopy

Caco-2 cells treated with DMSO, RSL3, or RSL3 + Fex for 24 h were harvested and fixed with 2.5% glutaraldehyde, followed by fixation with 1% OsO_4_. The samples were then washed, dehydrated with gradient ethanol, and embedded in araldite. Ultrathin sections were prepared for staining with uranyl acetate and lead citrate. Ultrastructural changes in the cells were examined via transmission electron microscopy (TEM) (JEOL JEM-1200EX, Tokyo, Japan).

### RNA sequence

The distal portions of murine colons were collected from the DSS-induced colitis (DSS group) and Fex-treated groups (Fex group) (*n* = 5 in each group) and subjected to BGI (Beijing Genomic Institute) for RNA sequencing. The RNA quality was evaluated with an Agilent 2100 Bioanalyzer (Agilent RNA 6000 Nano Kit). Library construction was performed according to the protocol provided for the BGISEQ-500 platform, and the libraries were sequenced on the BGISEQ-500 platform. Analysis was performed using the BGI online analysis platform (Dr. Tom).

### Lentiviral vectors and plasmid transfection

The phU6-EGFP-shRNA-FXR and phU6-EGFP-shRNA-SLC7A11 lentiviral vectors and their control vectors were used to inhibit FXR and SLC7A11 expression. FXR- and GPX4-overexpressing lentiviruses were used to overexpress Flag-FXR and Flag-GPX4, respectively, in vitro. All lentiviral vectors were provided by GeneChem Co., Ltd. (Shanghai, China). The Myc-GPX4 and HA-OTUB1 plasmids were produced by Tsingke Biotech Co., Ltd. (Beijing, China). SiRNAs targeting OTUB1 and scrambled negative control RNAs were purchased from GenePharma (China). Lipo8000 transfection reagent was purchased from Beyotime Biotechnology (Shanghai, China). All transfections were conducted following the manufacturer’s protocol.

### Quantitative chromatin Immunoprecipitation (qChIP)

An EZ-ChIP Kit (Millipore, Bedford, MA, USA) was used to perform a qChIP assay following the manufacturer’s protocol as described previously (Yu et al. 2019). Briefly, an anti-FXR antibody (#72105, Cell Signaling Technology, Inc.) and an IgG negative control antibody were added to precipitate the chromatin‒protein mixture. Specific primers were used to amplify DNA fragments with real-time PCR. The primers used are listed in Supplementary Table 1.

### Luciferase reporter assay

Fragments of the SLC7A11 5′ flanking sequence were cloned and inserted into the pGL3.0 Basic Vector (Promega, Madison, WI, USA) to generate the SLC7A11 promoter. Plasmid encoding the firefly luciferase reporter for the SLC7A11 promoter and the pTK-RL plasmid were cotransfected into cells. The subsequent detailed process was executed as described previously (Yu et al. 2019).

### Co-IP and mass spectrometry

Co-IP was performed as previously described (Zhao et al. 2024). Briefly, 1 × 10^7 cells were lysed for 30 min using Cell Lysis Buffer for western blotting and IP (P0013, Beyotime, Shanghai, China) premixed with PMSF and phosphatase inhibitors. Following centrifugation, the supernatant was collected and combined with an IP-grade antibody. The mixture was then incubated overnight on a shaker at 4 °C. The next day, agarose was added to the mixture, which was subsequently allowed to incubate for 4 h. The protein‒protein‒agarose complexes were subsequently precipitated via centrifugation and washed three times. Finally, the supernatant was discarded, and the Co-IP products were harvested. In addition to the Flag antibody (F1804, Sigma‒Aldrich) and IgG control antibody (30000-0-AP, Proteintech Group, Inc.), the antibodies utilized for Co-IP are listed in the “western blotting analysis” section. For the Co-IP assay, Co-IP products were boiled with loading buffer and analyzed via western blotting. For the mass spectrometry assay, the Co-IP products were analyzed using a Thermo Scientific Q Exactive mass spectrometer by Fitgene Biotechnology Co., Ltd. (Guangzhou, China).

### Coomassie blue staining

Co-IP was performed as described above, and the protein products of Co-IP were subsequently subjected to SDS–PAGE. The gel was subsequently stained with a Coomassie Blue Staining Kit (Beyotime, Shanghai, China). All procedures were performed following the instructions of the manufacturer.

### Statistical analysis

The measurement data are presented as the means ± SEMs. Differences between groups were identified with Student’s t test or ANOVA. Statistical analysis of the data in the present study was implemented using SPSS 22.0 software (SPSS Inc., Chicago, IL, USA). Statistical significance was established at *p* < 0.05. All experiments were performed in triplicate independently.

## Results

### FXR expression is impaired in ulcerative colitis

**Fig. 1:**
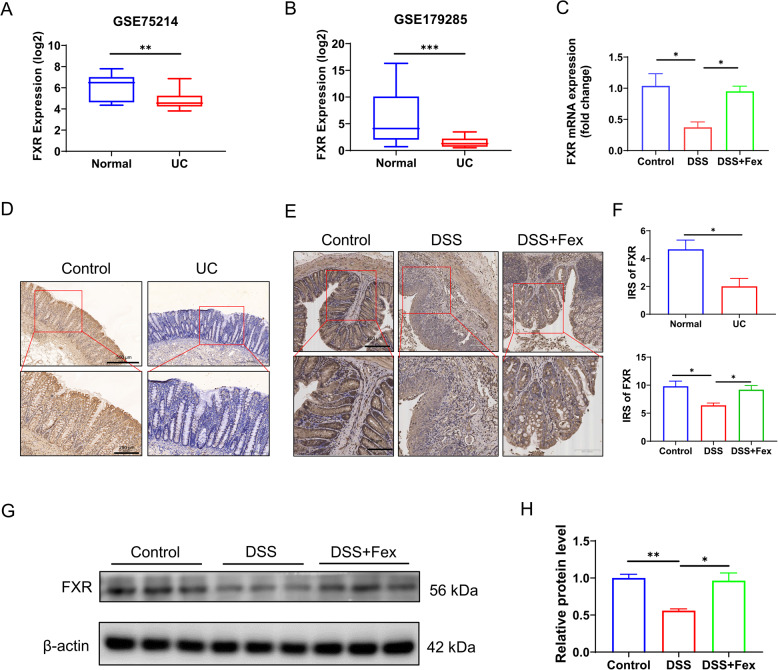
The expression level of FXR is impaired in ulcerative colitis. **A**,** B **Analysis of the FXR level via GSE75214 and GSE179285 in the GEO database revealed a change in FXR in patients with UC. **C** qRT‒PCR of colonic tissues from mice revealed changes in the mRNA level of FXR. β-actin was used as a loading control (*n* = 5 per group). **D** Representative images of IHC staining of FXR in colonic tissues from normal individuals and patients with UC. **E** Representative images of IHC results showing the change in FXR in mice with colitis and the effect of Fex treatment on FXR expression (*n* = 5 per group). **F** Immunoreactivity scores (IRSs) of IHC for FXR in humans (**D**) and murine colitis model mice (**E**). **G**,** H** western blotting bands (**G**) and corresponding quantitative data (normalized to β-actin) obtained via Image **J** (**H**) were used to evaluate FXR in colon tissues from the murine model (*n* = 5 per group). * *p* < 0.05; ** *p* < 0.01; ***p < 0.001

**Fig. 2:**
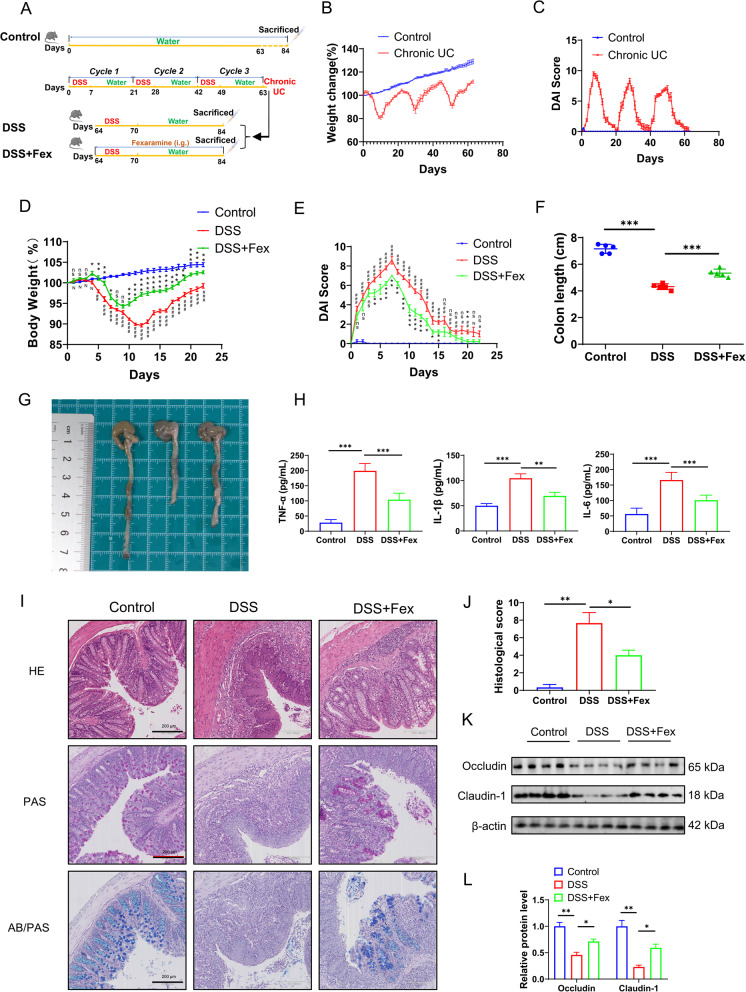
FXR activation ameliorates DSS-induced colitis. **A** Schematic of the process of chronic colitis induction and subsequent treatment. The experiment comprised three groups: the control, DSS, and DSS + Fex groups. The control group received regular water throughout. Chronic colitis (chronic UC) models were established through three cycles of 1 week of DSS treatment followed by 2 weeks of regular water. After the establishment of chronic UC, the DSS group subsequently continued with another cycle, whereas the DSS + Fex group received another cycle and additional Fex treatment (*n* = 5 per group). **B**,** C** Changes in body weight and DAI score during the induction of chronic UC. **D**,** E** Effects of vehicle (DSS group) or Fex (DSS + Fex group) on body weight (**D**) and DAI score (**E**) after the induction of chronic colitis. **F**,** G** Comparison of colon length and representative colon samples from mice subjected to different treatments. **H** The levels of inflammatory factors, including TNF-α, IL-1β, and IL-6, in murine serum were detected with the ELISA. **I**,** J** Evaluation of histological changes in the murine colon via **H** & **E**, PAS, and AB-PAS staining. **K**, **L** The expression levels of the tight junction proteins occludin and claudin-1 in colonic tissues were evaluated via western blotting (results from four mice per group are shown).* *p* < 0.05; ** *p* < 0.01; ***p < 0.001

First, the GEO database was used to investigate the expression of FXR (NR1H4) in patients with IBD. GSE75214 (colonic tissues with active inflammation from 74 patients with UC and normal colonic tissues from 11 control patients) and GSE179285 (colonic tissues with active inflammation from 23 patients with UC and normal colonic tissues from 31 control patients) revealed that the expression of FXR is downregulated in UC (Fig. 1A-B). In line with the above findings, local IHC analysis of colonic tissues revealed lower FXR in patients with UC than in control group (Fig. 1D and F). To investigate the pathogenesis of IBD and explore novel therapeutic approaches, various animal models have been established, among which dextran sodium sulfate (DSS)-induced colitis is widely employed as an ideal model for human UC. The chronic DSS-induced colitis model mimics the recurrent episodes characteristic of human UC. Thus, a chronic colitis model was constructed (Fig. 2A). Consistently, the results of qRT‒PCR (Fig. 1C), IHC (Fig. 1E and F), and western blotting (Fig. 1G H) demonstrated the compromised expression of FXR in colonic tissues. In addition, we constructed an acute colitis animal model by administering various concentrations of DSS for 7 days. As expected, higher concentrations of DSS corresponded to more severe colitis (Figure S1C-F). More severe colitis was associated with a lower FXR level (Figure S1A-B), indicating that FXR might also be involved in acute colitis.

### FXR activation mitigates DSS-induced colitis

To evaluate the effect of intestinal FXR activation on colitis, chronic colitis was induced with DSS to simulate human colitis in the first step, and then, the mice with chronic colitis were treated with vehicle (DSS group) or the intestinal-specific FXR agonist Fex (DSS + Fex group). A schematic diagram of the animal experimental procedure is depicted in Fig. 2A. Changes in weight and the DAI score, crucial indicators of the severity of colitis, confirmed that the chronic colitis model was successfully established (Fig. 2B-C; Figure S2A-S2C). The role of FXR activation in UC was subsequently investigated. The results revealed that treatment with Fex clearly restored the level of FXR in DSS-induced colitis (Fig. 1E and H). Moreover, Fex distinctly attenuated DSS-induced colitis in terms of body weight loss, DAI score, and shortened colon length (Fig. 2D-G). In addition, we evaluated the levels of the proinflammatory cytokines TNF-α, IL-1β, and IL-6, which are considered indicators of the extent of inflammation, in murine serum (Fig. 2H) (Liu et al. 2020). The severity of colon histological damage in the Fex-treated group was also lower than that in the DSS group, as shown in Fig. 2I and J. The tight junction proteins occludin and claudin-1 are representative hallmarks of the integrity of the intestinal barrier (Kumar et al. 2021; Sharma et al. 2018). Our studies revealed that Fex significantly protected the intestinal barrier (Fig. 2K and L). In summary, an FXR agonist alleviates intestinal inflammation and protects against tissue damage in mice with DSS-induced chronic colitis.

### FXR activation inhibits ferroptosis in intestinal epithelial cells in DSS-induced colitis

**Fig. 3:**
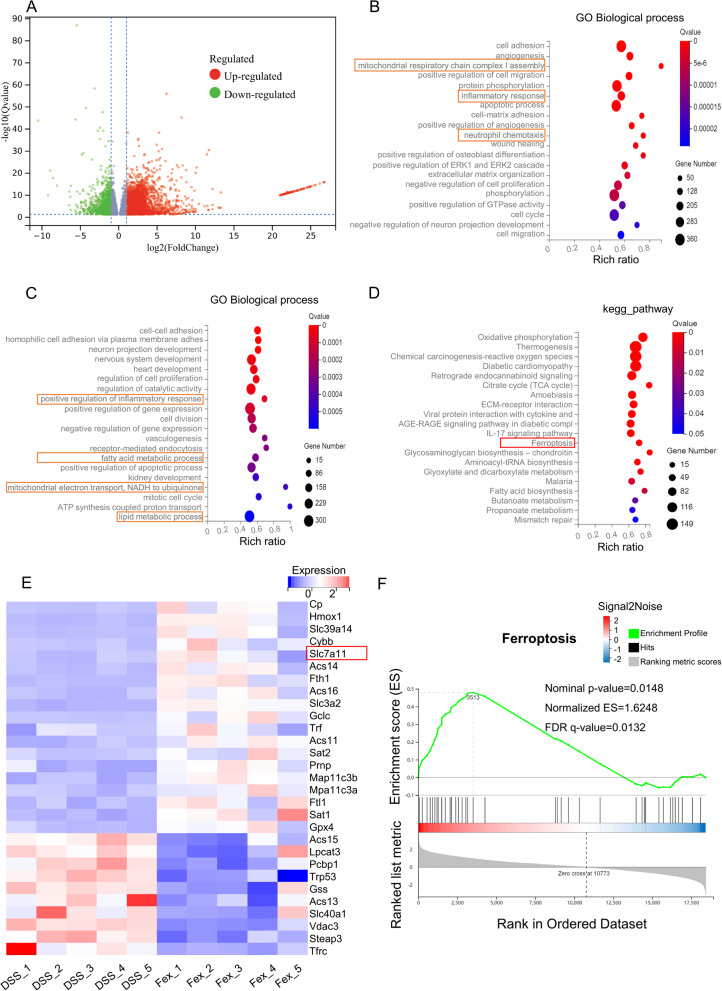
RNA-sequencing data of colonic tissues from DSS-induced colitis mice treated with vehicle (DSS group) or Fex (DSS + Fex group). **A** DEGs (differentially expressed genes,|log2FC|≥1, Q value ≤ 0.05) between the two groups are shown in volcano plots. **B**,** C** Bubble diagram of the GO enrichment analysis results showing the top 1–20 (B) and top 21–40 (**C**) biological processes. **D**,** E** KEGG pathway enrichment analysis of the DEGs revealed the top 1–20 (**D**) and top 21–40 (**E**) pathways. **F** Ferroptosis-related genes among the DEGs are shown in a heatmap. (G) GSEA was performed based on the GSEA_KEGG_PATHWAY database

**Fig. 4:**
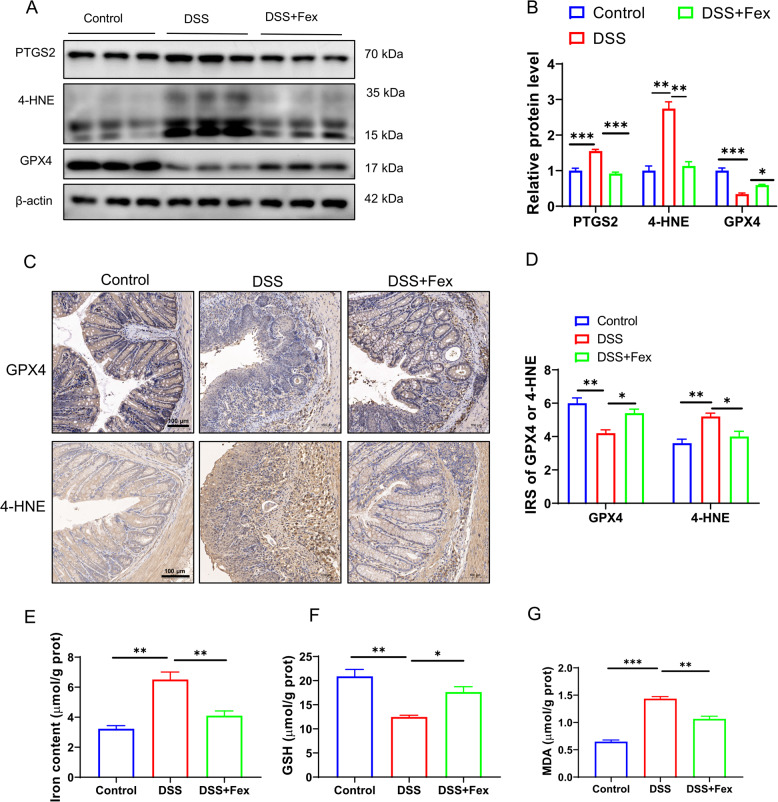
FXR activation inhibits ferroptosis in intestinal epithelial cells from patients with DSS-induced colitis. **A**,** B** western blotting images and normalized data of PTGS2 and GPX4 expression in the colonic tissues of the mice in different groups (*n* = 5 per group). β-actin was used as a loading control. **C**,** D** IHC of GPX4 and 4-HNE in colonic tissues from different groups. **E-G** Total iron (**E**), GSH (**F**), and MDA (**G**) concentrations in colonic homogenates were measured. * *p* < 0.05; ** *p* < 0.01; ***p < 0.001

Ferroptosis of intestinal epithelial cells is involved in colitis, and the restriction of ferroptosis is conducive to the remission of DSS-induced colitis (Tang et al. 2021; Xu et al. 2020, 2021b). Nevertheless, it is still unclear whether the activation of FXR can alleviate ferroptosis in patients with colitis. Hence, colonic tissues from the DSS and DSS + Fex groups were subjected to RNA sequencing (*n* = 5 in each group). The analysis identified 5027 upregulated differentially expressed genes (DEGs,|log2FC|≥1, Q value ≤ 0.05) and 3783 downregulated DEGs (Fig. 3A). The GO analysis revealed that the DEGs were enriched in biological processes related to “inflammatory”, “mitochondria”, “fatty acid metabolism”, and “lipid metabolism”, which are associated with colitis and ferroptosis (Fig. 3B, top 1–20; Fig. 3C, top 21–40). In addition, KEGG pathway analysis indicated that Fex treatment affected genes related to ROS and ferroptosis (Fig. 3D, top 1–20 pathways). A heatmap of the ferroptosis-related genes was constructed (Fig. 3E). GSEA further revealed that the ferroptosis pathway was significantly enriched (Fig. 3F). Consistently, our subsequent experiments revealed that ferroptosis was involved in DSS-induced colitis, as shown in Fig. 4A-G. Fex treatment not only increased the FXR protein level (Fig. 1G) but also reversed the impaired GPX4 expression and mitigated the increased PTGS2 and 4-HNE protein levels (symbols reflecting the extent of ferroptosis) induced by DSS (Fig. 4A and B). Furthermore, the expression profiles of GPX4 and 4-HNE were evaluated via IHC (Fig. 4C and D), which revealed consistent results. Aberrant accumulation of Fe increased the risk of ferroptosis. The Fe concentration assay revealed that DSS increased the content of Fe, whereas Fex application significantly inhibited this effect (Fig. 4E). As an indispensable cofactor for the antiferroptotic role of GPX4, GSH was also improved by Fex in DSS-induced colitis (Fig. 4F). The level of MDA, an oxidized lipid metabolite involved in ferroptosis, was increased following DSS treatment but could be reversed with additional treatment with Fex (Fig. 4G). Accordingly, FXR activation by Fex attenuated ferroptosis in DSS-induced colitis.

In addition, to further confirm that FXR modulates ferroptosis in colitis, FXR knockout (FXR^-/-^) mice were used. As shown in Fig. S3, after FXR knockout, DSS-induced colitis was significantly aggravated (Fig. S3A-S3D), accompanied by a notable exacerbation of ferroptosis severity (Fig. S3E-S3F). Moreover, FXR knockout also negated the ability of Fex to alleviate ferroptosis (Fig. S3E-S3F).

### FXR activation restrains ferroptosis in Caco-2 cells

**Fig. 5:**
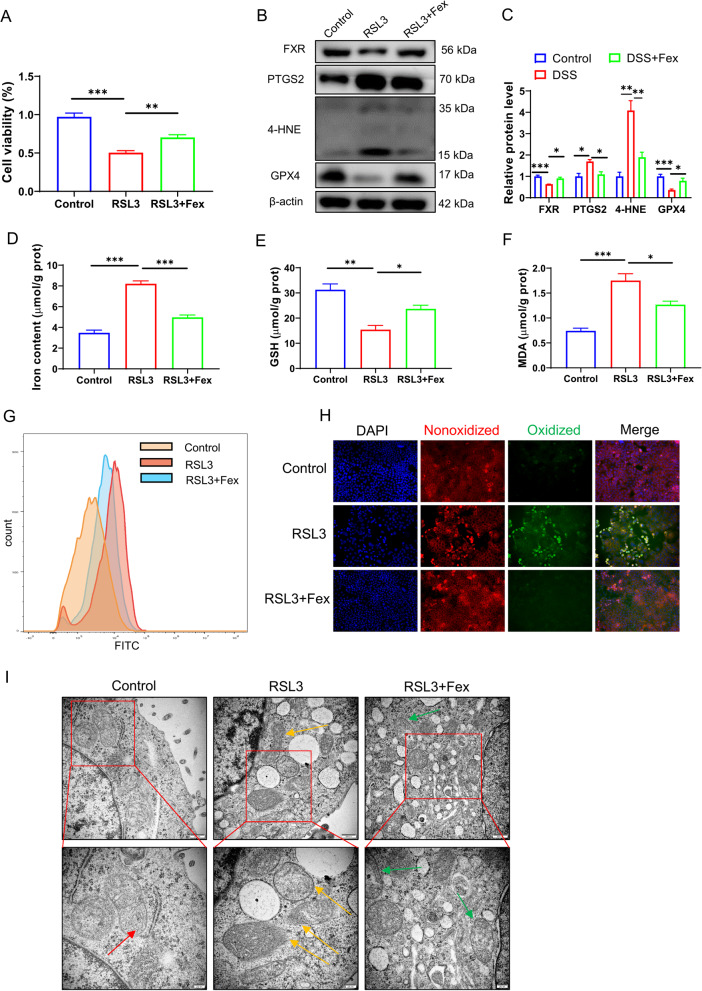
FXR activation restrains ferroptosis in Caco-2 cells. **A** RSL3 was utilized to induce ferroptosis in vitro. A cell viability assay was performed to determine the role of Fex in Caco-2 cells. **B**,** C** western blotting (**B**) and quantification of the immunoblots (**C**) were performed to compare the protein levels of GPX4 and PTGS2 in Caco-2 cells. **D-F** The contents of Fe (**D**), GSH (**E**), and MDA (**F**) in Caco-2 cells treated with DMSO, RLS3, or RSL3 + Fex were monitored using specific assay kits. **G** ROS levels were measured in Caco-2 cells via flow cytometry (FCM). **H** Lipid peroxidation of Caco-2 cells marked with C11 BIODIPY 581/591 was detected with a fluorescence microscope. **I** TEM scanning of Caco-2 cells revealed ferroptosis-specific changes in mitochondria in response to different treatments. The red arrows indicate normal mitochondria with proper membrane density and clear mitochondrial cristae. Yellow arrows indicate increased membrane density, the disappearance of mitochondrial cristae, and the rupture of the mitochondrial outer membrane. Green arrows indicate improved mitochondrial morphology. * *p* < 0.05; ** *p* < 0.01; ***p < 0.001

RSL3 is a well-known GPX4 inhibitor that triggers ferroptosis. To investigate the effects of an FXR agonist on ferroptosis in vitro, we induced ferroptosis with RSL3 in Caco-2 cells, which are derived from intestinal epithelial cells (IECs) and are commonly used as an IEC model in vitro. As shown in Fig. 5A, treatment with RSL3 significantly suppressed cell viability. Nevertheless, the application of the FXR agonist Fex improved the cell viability inhibited by RSL3. The level of GPX4 was reduced and that of PTGS2 was increased following RSL3 treatment, effects that could be partly rescued by Fex treatment (Fig. 5B and C). In addition, the concentrations of total iron (Fig. 5D), GSH (Fig. 5E), and MDA (Fig. 5F) were detected in different groups to evaluate the extent of ferroptosis, which highlighted the protective role of Fex in RSL3-induced ferroptosis. The ROS level measured with DCFH-DA via flow cytometry (FCM) (Fig. 5G) revealed that Fex alleviated the increase in the ROS level induced by RSL3. Lipid peroxidation, the executioner of cellular membrane destruction in ferroptosis, was evaluated with C11 BODIPY 581/591 by fluorescence microscopy. The results of C11 BODIPY 581/591 staining were consistent with these findings (Fig. 5H). The hallmark characteristic of ferroptosis is morphological changes in mitochondria. TEM confirmed that treatment with Fex alleviated mitochondrial damage (Fig. 5I). Taken together, these results indicate that an FXR agonist restrains ferroptosis induced by RSL3 in vitro.

### FXR directly transcriptionally upregulates SLC7A11 expression

System Xc–GSH-GPX4, GCH1-BH_4_, and the AIFM2- CoQ10 axis are ubiquitous in defending against ferroptosis, as mentioned above. To elucidate the mechanism by which FXR modulates ferroptosis, we reviewed the DEGs identified from the RNA-seq data. Among the ferroptosis-related genes, SLC7A11 was increased, with a considerable fold change (log2FC = 2.39, Q value = 3.88E‒05), and it was also one of the key subunits of System Xc−. As a transcription factor, FXR is involved in the modulation of multiple genes. Thus, we used the JASPAR database to evaluate whether FXR is capable of binding to the promoter region of SLC7A11. The AGGTCAgtTGAGCA sequence was subsequently found in the promoter region of the SLC7A11 gene, which may be an imperfect inverted repeat motif, IR2 (inverted repeat-2). IR2 can serve as a binding site for the FXR response element (Li et al. 2008).

**Fig. 6:**
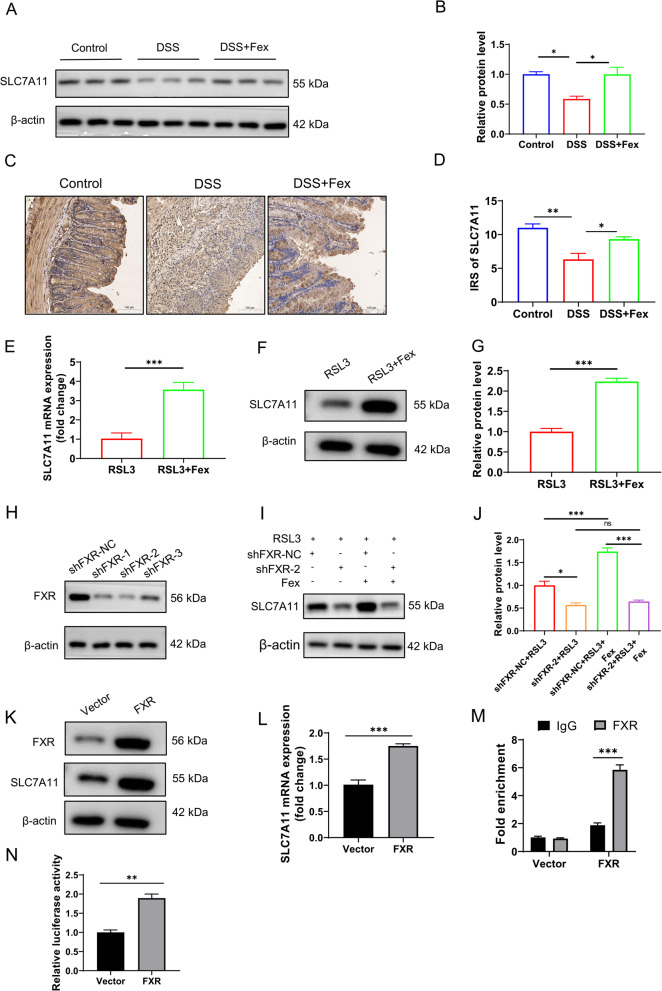
FXR directly transcriptionally upregulates SLC7A11 expression. **A** western blotting was used to detect SLC7A11 in colonic tissues from mice (*n* = 5 per group). **B** Quantification of the data in (**A**) using ImageJ software, with β-actin used as a loading control. **C**,** D** SLC7A11 levels were evaluated by IHC in mice. **E-G** Effects of Fex treatment on SLC7A11 mRNA (**E**) and protein levels (**F **and **G**) in RSL3-treated Caco-2 cells. **H** Verification of FXR knockdown in Caco-2 cells. **I**,** J** Effects of FXR knockdown and Fex treatment on SLC7A11 expression in Caco-2 cells stimulated with RSL3. **K**,** L** The effect of FXR on SLC7A11 protein expression was investigated by overexpressing FXR in Caco-2 cells. **M** qCHIP was used to detect the binding between FXR and the promoter region of SLC7A11. **N** A luciferase reporter assay was employed to evaluate the transcriptional activity of FXR to SLC7A11. * *p* < 0.05; ** *p* < 0.01; ***p < 0.001

To investigate the relationship between FXR and SLC7A11, western blotting was performed. The results revealed that DSS challenge impaired SLC7A11 in DSS-induced colitis, whereas Fex treatment significantly elevated SLC7A11 levels (Fig. 6A and B). IHC and qRT‒PCR revealed accordant changes in murine colonic tissues (Fig. 6C and E). In in vitro experiments, Fex treatment following RSL3 challenge increased SLC7A11 expression compared with the RSL3 group that did not receive Fex (Fig. 6F and G). Next, we knocked down the expression of FXR in Caco-2 cells, as shown in Fig. 5H. Compared with shFXR-NC, knockdown of FXR decreased the expression of SLC7A11 upon RSL3 challenge (Fig. 6I). Moreover, knockdown of FXR counteracted the promoting role of Fex in SLC7A11 cells (Fig. 6I and J). In addition, FXR overexpression led to elevated SLC7A11 mRNA and protein levels in Caco-2 cells (Fig. 6K and L). Subsequently, ChIP‒PCR and luciferase reporter assays were performed. The results confirmed the role of FXR in promoting SLC7A11 transcription (Fig. 6M and N).

### FXR activation protects intestinal epithelial cells from ferroptosis through SLC7A11

**Fig. 7:**
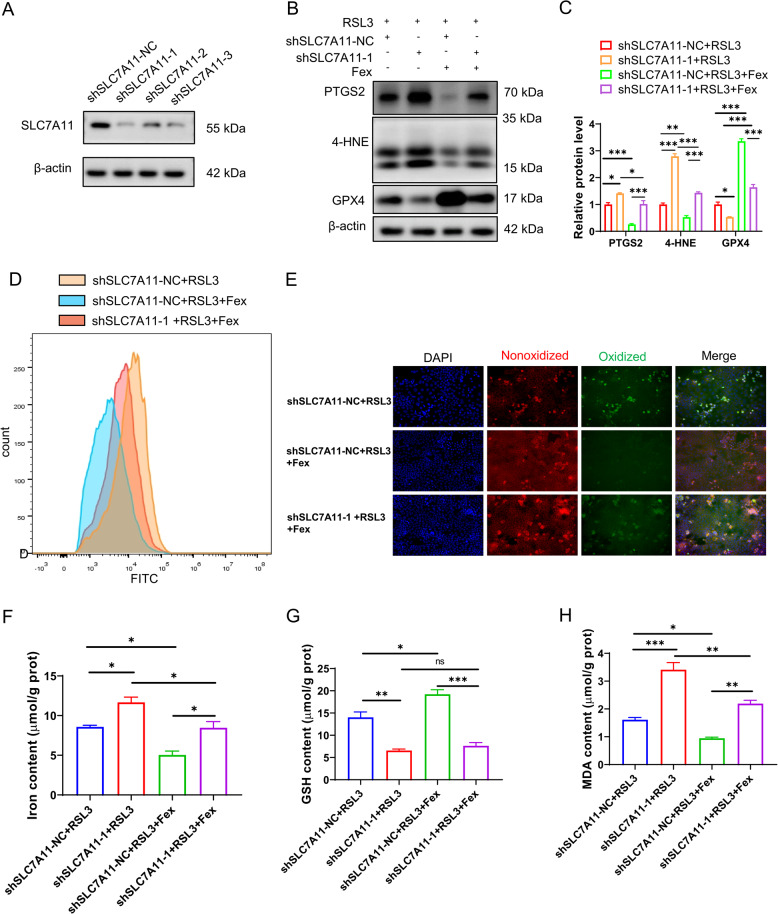
FXR activation protects intestinal epithelial cells from ferroptosis through SLC7A11. **A** The effect of SLC7A11 knockdown was verified by western blotting in Caco-2 cells. **B**,** C** Effects of SLC7A11 knockdown and Fex treatment on ferroptosis in Caco-2 cells stimulated with RSL3. **D** ROS levels were evaluated by DCFH-DA via FCM in Caco-2 cells subjected to specific treatments. **E** Lipid peroxidation of Caco-2 cells marked with C11 BIODIPY 581/591 was detected with a fluorescence microscope. **F-H** Iron (**F**), GSH (**G**), and MDA (**H**) contents in Caco-2 cells were monitored with specific assay kits. * *p* < 0.05; ** *p* < 0.01; ***p < 0.001

To further confirm that the FXR agonist protects intestinal epithelial cells from ferroptosis via transactivation of SLC7A11, we inhibited SLC7A11 via shRNA in vitro. Western blotting was used to identify the effect of SLC7A11 knockdown (Fig. 7A). The levels of PTGS2, 4-HNE, and GPX4 (Fig. 7B and C) were evaluated by western blotting to determine the extent of ferroptosis. The results showed that SLC7A11 is essential for the protective function of FXR. Consistently, the ROS levels (Fig. 7D) determined by DCFH-DA and lipid peroxidation with C11 BIODIPY 581/591 (Fig. 7E) were similar. Compared with those in the NC group, total Fe (Fig. 7F) and MDA (Fig. 7G) were subsequently increased, whereas the concentration of GSH (Fig. 7H) was decreased in shSLC7A11-transfected Caco-2 cells. Overall, the protective role of the FXR agonist in ferroptosis was restrained upon SLC7A11 depletion.

### FXR agonists suppressed ferroptosis via the deubiquitination of GPX4

**Fig. 8:**
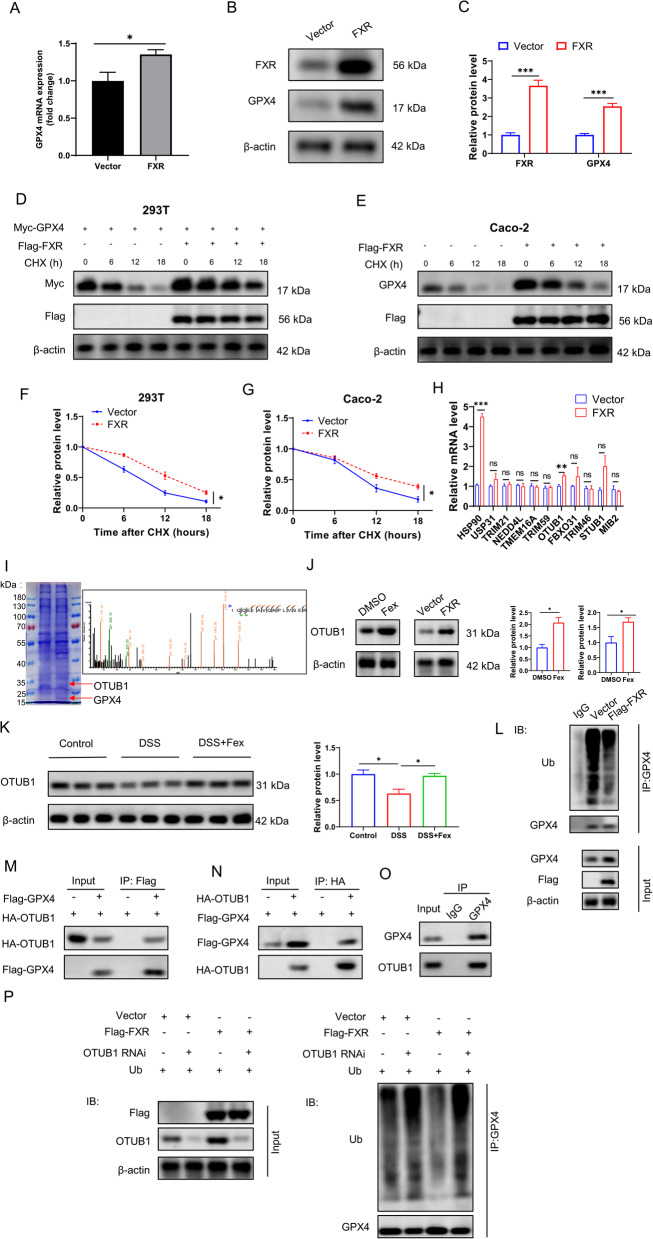
FXR agonist protects intestinal epithelial cells from ferroptosis through SLC7A11. **A** The mRNA levels of GPX4 were measured via qRT‒PCR following FXR overexpression. **B**, **C** Protein levels of GPX4 were detected after FXR overexpression. **D**, **F** Transfection of GPX4 and FXR plasmids in 293 T cells, followed by CHX assay to assess the level of exogenous GPX4. Cells were treated with CHX (20 µg/ml) and harvested at the indicated time points. **E**, **G** Overexpression of FXR in Caco-2 cells was followed by CHX treatment to evaluate the protein stability of endogenous GPX4. Cells were treated with CHX (20 µg/ml) and harvested at the indicated time points. **H** mRNA levels of potential GPX4 ubiquitination regulatory genes were examined after FXR overexpression. **I** Co-IP was performed with an anti-Flag antibody in Caco-2 cells transfected with vector or Flag-GPX4. The products of Co-IP were subjected to Coomassie blue staining (left panel) and mass spectrometry analysis. Mass spectrometry peak graph showing the presence of OTUB1 peptide segments in the CoIP products of Caco-2 cells transfected with GPX4 (right panel). **J** Protein levels of OTUB1 were measured after RSL3 stimulation, with the addition of Fex (left panel) or FXR overexpression (right panel). **K** OTUB1 protein levels in DSS-induced colitis were assessed. **L** Ubiquitination levels of GPX4 were measured after FXR overexpression. **M** Flag-GPX4 and HA-OTUB1 plasmids were transfected into 293 T cells, followed by IP with an anti-Flag antibody. **N** HA-GPX4 and Flag-OTUB1 plasmids were transfected into 293 T cells, followed by IP with an anti-Flag antibody. **O** IP was performed in Caco-2 cells with IgG or an anti-GPX4 antibody. **P** Measurement of GPX4 protein ubiquitination levels after transfection of the respective plasmids in 293 T cells. For the ubiquitination detection experiment, MG132 (20 µM) was added to the medium 6 h before the cells were collected

Interestingly, as shown in Fig. 7, SLC7A11 knockout significantly attenuated the antiferroptotic effect of FXR agonists but did not completely abolish it. Consequently, we postulated that FXR might mitigate ferroptosis through alternative mechanisms. Notably, GPX4 not only serves as a marker for ferroptosis but also plays a crucial role in the anti-ferroptotic process. In Figs. 4 and 5, we noticed a substantial increase in GPX4 expression following FXR activation, yet it remained unclear whether FXR directly promoted the elevation of GPX4 or if GPX4 underwent compensatory upregulation on its own. As shown in Fig. 7B, even after SLC7A11 was knocked down, Fex still increased the expression level of GPX4 (the fourth lane compared with the second lane). Previous studies have suggested that FXR can markedly increase GPX4 mRNA levels (Tschuck et al. 2023); therefore, we examined the mRNA levels of GPX4 after FXR overexpression. As shown in Fig. 8A, only a slight increase in GPX4 transcription was observed following FXR overexpression, which was far less than the change in protein levels (Fig. 8B and C). Hence, we hypothesized that FXR might regulate GPX4 levels by influencing its protein stability. Through cycloheximide (CHX) experiments, we found that FXR overexpression significantly increased the stability of exogenous GPX4 in 293 T cells (Fig. 8D and F) and of endogenous GPX4 in Caco2 cells (Fig. 8E and G). Ubiquitination is a primary mechanism for protein degradation, and previous studies have shown that several genes can affect GPX4 protein levels by altering its ubiquitination status. Therefore, we examined the mRNA levels of common GPX4 ubiquitination regulatory proteins reported in the literature, including HSP90 (Zhou et al. 2023), USP31 (Yao et al. 2023), TRIM21 (Sun et al. 2023a), NEDD4L (Tang et al. 2023), TMEM16A (Guo et al. 2022), TRIM59 (ZHang et al. 2023), OTUB1 (Li et al. 2023), FBXO31 (Zhu et al. 2022), TRIM46 (ZHang et al. 2021), STUB1 (Sun et al. 2023b), and MIB2 (Zhao et al. 2021). As depicted in Fig. 8H, HSP90 and OTUB1 exhibited significant alterations following FXR overexpression. Additionally, through coimmunoprecipitation (Co-IP) coupled with mass spectrometry analysis, we identified potential proteins that interact with GPX4. Coomassie blue staining and mass spectrometry analysis suggested that OTUB1 might interact with GPX4 (Fig. 8I). Subsequently, we separately examined the effects of Fex treatment and FXR overexpression on OTUB1, revealing a notable increase in OTUB1 protein levels (Fig. 8J). By examining intestinal tissues from DSS-induced colitis mice, we found a significant increase in OTUB1 protein levels in the Fex treatment group relative to those in the DSS model group. Next, we assessed the impact of FXR on the ubiquitination level of the GPX4 protein. As shown in Fig. 8L, FXR overexpression markedly reduced the level of ubiquitinated GPX4. Through Co-IP experiments, we found that OTUB1 could interact with the GPX4 protein (Fig. 8M-O). Furthermore, knockdown of OTUB1 significantly abolished the ability of FXR to reduce GPX4 ubiquitination. Taken together, these findings indicate that FXR can alleviate GPX4 ubiquitination levels and increase GPX4 content by upregulating the deubiquitinase OTUB1.

## Discussion

In this study, we revealed that the activation of FXR confined DSS-induced colitis via various mechanisms, including alleviating ferroptosis. As a transcription factor, FXR can induce gene transcription by directly binding to the FXR response element (FXRRE) in the promoter regions of target genes. Researchers have rarely investigated whether FXR modulates the transcription of ferroptosis-related genes in patients with colitis. In this study, SLC7A11 was identified as a novel downstream gene of FXR. Additionally, we found that FXR could alleviate ferroptosis by modulating GPX4 ubiquitination. These findings may pave the way for the development of drugs that target FXR for the treatment of colitis.

The proliferation and death of IECs are accurately modulated under physiological conditions to maintain intestinal homeostasis. As previously mentioned, multiple types of PCD are involved in colitis, including but not limited to apoptosis (Kuo et al. 2019), pyroptosis (Yuan et al. 2018), necroptosis (Zhou et al. 2021), and autophagy (Larabi et al. 2020). The role of ferroptosis, a newly described form of cell death, in colitis has been highlighted recently. Ferroptosis of IECs leads to destruction of the gut barrier. As a result, the microbiota and toxins derived from the gut lumen are translocated to the gut wall, triggering an inflammatory cascade that may initiate or aggravate colitis. It has been reported that restricting the death of IECs may alleviate colitis (Shao et al. 2022). In the present study, we found that ferroptosis was associated with serious symptoms, including body weight loss, hematochezia, and diarrhea, in DSS-induced colitis, whereas the repression of ferroptosis by the activation of FXR was correlated with minor symptoms. In addition, a novel study revealed that ferroptosis in intestinal intraepithelial lymphocytes might also be involved in colitis (Panda et al. 2023). These results provide a new therapeutic strategy for treating colitis via the suppression of ferroptosis.

As a nuclear receptor, FXR is essential for the modulation of bile acid metabolism in the intestinal tract. A proper bile acid profile is involved in maintaining intestinal homeostasis by affecting the intestinal microbiota, intestinal mucosa, and inflammatory response. Bile acid signals are abnormal in patients with IBD (Zhou et al. 2022). Accordingly, it has been reported that FXR mRNA expression is significantly reduced in the inflamed colons of DSS-induced IBD mice and children with IBD (Negroni et al. 2020). In line with the above findings, we found that the mRNA level of FXR in UC tissues was lower than that in normal colonic tissues via analysis of the GEO database and tissues from mice with DSS-induced colitis. Furthermore, IHC of colonic sections from patients with UC and western blotting of DSS-induced murine colon tissues verified that the protein level of FXR is suppressed in colitis. These results highlight the compromised profile of FXR in the development of colitis.

Previous studies revealed that FXR is involved in various types of cell death, including pyroptosis (Guo et al. 2021), apoptosis (Girisa et al. 2021), and autophagy (Kim et al. 2021). The activation of FXR has also been proven to protect against mitochondrial dysfunction and relieve oxidative stress in nephropathy and hepatocytes (Lee et al. 2012; Wang et al. 2018). Miyazaki et al. reported that a novel FXR agonist, nelumal A, decreased oxidative damage in DSS-induced colitis (Miyazaki et al. 2021). Oxidative stress is associated with a variety of pathological processes, including ferroptosis (Zuo et al. 2022). This prompted FXR may be involved in ferroptosis. As expected, a novel study reported that FXR alleviated ferroptosis in cisplatin-induced acute kidney injury (Kim et al. 2022). Nevertheless, whether FXR regulates ferroptosis in colitis has not been elucidated. In the present study, we found that treatment with the intestine-restricted FXR agonist Fex increased the levels of GPX4 and GSH, whereas the Fe and MDA contents decreased in vivo and in vitro. As a hallmark of ferroptosis, these changes suggest that FXR may be involved in the regulation of ferroptosis. Further TEM examination confirmed the protective role of FXR in ferroptosis. Therefore, we provide novel information for further understanding the role of FXR in colitis.

IBD development is associated with abnormal mucosal immunity, an impaired intestinal epithelial barrier, and intestinal dysbacteriosis in populations with genetic susceptibility (Xavier and Podolsky 2007), and FXR was shown to play a protective role in these aspects. FXR activation not only prevented goblet cell loss and decreased epithelial permeability but also inhibited proinflammatory cytokine secretion in various primary human immune cell types and alleviated NF-kB-mediated immune responses in enterocytes (Gadaleta et al. 2011). Moreover, the FXR ligand GW4064 reduced bacterial translocation by upregulating several genes with antimicrobial properties, including iNOS, IL18, and Ang1 (Inagaki et al. 2006). Fex administration increased the abundance of SCFA-producing bacteria and led to reduced intestinal inflammation (Xu et al. 2021a). Intriguingly, Huo et al. reported that acyclic sesquiterpenoids secreted by *Candida metapsilosis* M2006B functioned as FXR agonists to alleviate colitis (Huo et al. 2022), resulting in crosstalk between FXR and the gut microbiota. The results of the present study further revealed a novel mechanism by which FXR ameliorates colitis by limiting ferroptosis. In view of the versatile role of FXR in colitis, FXR agonists could be valuable therapeutic agents.

Many previous studies have focused on the role of FXR in CRC. FXR levels are downregulated in CRC (Bailey et al. 2014). Research has shown that FXR is a potent inhibitor of tumor development both in vivo and in vitro (Zhou et al. 2022). Our previous study revealed that FXR plays a tumor-suppressor role in colorectal cancer (Yu et al. 2020). Research has demonstrated that targeting ferroptosis pathways is helpful for cancer therapy (Tong et al. 2022). Hence, the induction of ferroptosis by disrupting glutathione metabolism is considered a promising strategy for CRC treatment (Yan et al. 2022). Nonetheless, in the present study, we found that FXR could upregulate SLC7A11, indicating that FXR might be able to help tumor cells evade ferroptosis to some extent. This function of FXR seems paradoxical with its anti-CRC role described previously. The following viewpoints may provide insights into this question. On the one hand, FXR has pleiotropic functions in CRC, such as inducing pyroptosis (Guo et al. 2021), inhibiting the proliferation of CRC cells (Qiao et al. 2018), and regulating multiple metabolic processes (Huang et al. 2022). However, it was unclear which process was the leading function. On the other hand, ferroptosis seems to have dual functions in the development of tumors according to the specific environment. Ferroptosis-induced inflammatory reactions may potentiate the progression of tumors by releasing DAMPs (Dang et al. 2022). Thus, for some tumors, inducing ferroptosis may be detrimental. The heterogeneity of tumor cells may determine the predominant pathway of the intricate function of FXR. Detailed studies are needed to elucidate the relationship between FXR and ferroptosis in tumor development.

Kim et al. reported that FXR can directly bind to the ferroptosis-related genes Aifm2 (a crucial constituent of the AIFM2–CoQ10 axis), Ggt6, and Gsta4 in cisplatin-induced acute kidney injury (Kim et al. 2022). To elucidate the mechanism by which FXR regulates ferroptosis in colitis, we performed RNA sequencing and then focused on the subunit of System Xc, termed SLC7A11. SLC7A11 serves as a key factor in maintaining the homeostasis of cystine/glutamate metabolism by exchanging intracellular glutamate with extracellular cystine at a 1:1 ratio. Cystine is a rate-limiting precursor of GPX4-mediated detoxification. Blockade of SLC7A11 stalls the import of cystine, leading to a deficiency in GSH synthesis. As a result, lipid peroxides cannot be detoxified to avirulent lipid alcohols by GPX4, and ferroptosis occurs. Hence, numerous studies have focused on the regulatory effects of SLC7A11 on transcription, epigenetic modification, and posttranslational control. Activating transcription factor 4 (ATF4) and NRF2 serve as the two predominant factors that regulate SLC7A11 transcriptionally. Moreover, ATF4 (activating transcription factor 4) retarded ferroptosis via the upregulation of SLC7A11 (Chen et al. 2017). The accumulation of NRF2 also facilitates the transcription of SLC7A11, resulting in the remission of ferroptosis. p53 and ATF3 are considered the two primary negative transcriptional regulatory factors of SLC7A11, and both can induce ferroptosis (Jiang et al. 2015; Wang et al. 2020). In the present study, ChIP analysis and a luciferase reporter assay indicated that FXR bound directly to the promoter of SLC7A11, leading to the attenuation of ferroptosis. Knockdown of SLC7A11 by shRNA crippled the protective role of Fex against ferroptosis in RSL3-challenged Caco-2 cells. Accordingly, we identified a novel mechanism by which SLC7A11 is regulated by FXR transactivation. Notably, SLC7A11 knockdown abolished the Fex-induced increase in the GSH level, whereas the protective effect of Fex was not completely eliminated (Fig. 6F-H), indicating that other mechanisms by which Fex alleviates ferroptosis in IECs may exist. Our further investigations revealed that FXR can indirectly influence ferroptosis by altering the ubiquitination level of GPX4.

Similarly, there are disadvantages in our study. As most of our work was based on cell lines and animal models, the specific mechanism in patients needs to be verified in further studies.

Colitis is a chronic and relapsing-remitting digestive disease with worldwide incidence. Although many studies have focused on IBD for decades, unfortunately, no highly effective treatments have been identified thus far. This study has opened broad research avenues for better understanding the role of FXR in colitis and has revealed that FXR can inhibit ferroptosis in colitis through dual mechanisms.

## Conclusions

In conclusion, FXR is compromised in colitis, and restoring FXR has a protective effect on colitis by suppressing ferroptosis via direct transactivation of SLC7A11 and stabilizing GPX4. Therefore, these findings may provide new insights into and options for the treatment of colitis.

## Supplementary Information

Supplementary Material 1

## Data Availability

No datasets were generated or analysed during the current study.
