# Supplementary material for: FXR acts as a therapeutic target for ulcerative colitis via suppressing ferroptosis
Source: Mol Med. 2025 Jul 18;31:258. doi: 10.1186/s10020-025-01305-3 (PMC12273381; doi:10.1186/s10020-025-01305-3)
Supplement: Supplementary file 1 — Supplementary Material 1 [file 10020_2025_1305_MOESM1_ESM.docx]

**Supplementary figures**





**Figure S1. The level of intestinal FXR is associated with the severity of colitis.**

(A, B) Immunohistochemistry (IHC) analysis showed that the level of FXR is negatively correlated with DSS concentration in the murine colitis model. (C) Colon length in mice treated with various concentrations of DSS. (D) Disease activity index (DAI) scores of different groups during the colitis model development. (E) Representative images of the colon from different experimental groups. n=5 per group.



**Figure. S2 Specific scores of different aspects during animal experiments.**

(A-C) Specific scoring for body weight (A), fecal blood (B), and stool consistency (C) during the construction of the chronic colitis model. (D-F) Mice were treated with water (Control group), DSS (DSS group), or DSS plus Fex (DSS+Fex group). Body weight (D), fecal blood (E), and stool consistency (F) were scored. n=5 per group

**

Figure S3. Acute colitis models in wild type (WT) and FXR knockout (FXR-/-) mice.**

(A) Acute colitis models were established in WT and FXR-/- mice through 7 days of DSS induction, with or without Fex treatment. The colons of mice were excised intact for photography. (B) Analysis of colon lengths in mice from different groups .(C-D) Percentage change in body weight (C) and DAI scores (D) during DSS-induced acute colitis in mice. (E-F) Total protein extracted from colon tissues of mice from different groups was subjected to Western blotting to detect ferroptosis markers. n=5 per group
